# Proposition and Real-Time Implementation of an Energy-Aware Routing Protocol for a Software Defined Wireless Sensor Network

**DOI:** 10.3390/s19122739

**Published:** 2019-06-18

**Authors:** Muhammad Usman Younus, Saif ul Islam, Sung Won Kim

**Affiliations:** 1 Institut de Recherche en Informatique de Toulouse (IRIT), Université Paul Sabatier, 31400 Toulouse, France; muhammad.younus@irit.fr; 2 Department of Electrical and Computer Engineering, COMSATS University, Islamabad 44000, Pakistan; 3 Department of Computer Science, Dr. A. Q. Khan Institute of Computer Science and Information Technology, Rawalpindi 47000, Pakistan; saiflu2004@gmail.com; 4 Department of Information and Communication Engineering, Yeungnam University, Gyeongsan 38542, Korea

**Keywords:** SDN, WSN, SDWSN, energy-aware routing

## Abstract

A wireless sensor network (WSN) has achieved significant importance in tracking different physical or environmental conditions using wireless sensor nodes. Such types of networks are used in various applications including smart cities, smart building, military target tracking and surveillance, natural disaster relief, and smart homes. However, the limited power capacity of sensor nodes is considered a major issue that hampers the performance of a WSN. A plethora of research has been conducted to reduce the energy consumption of sensor nodes in traditional WSN, however the limited functional capability of such networks is the main constraint in designing sophisticated and dynamic solutions. Given this, software defined networking (SDN) has revolutionized traditional networks by providing a programmable and flexible framework. Therefore, SDN concepts can be utilized in designing energy-efficient WSN solutions. In this paper, we exploit SDN capabilities to conserve energy consumption in a traditional WSN. To achieve this, an energy-aware multihop routing protocol (named EASDN) is proposed for software defined wireless sensor network (SDWSN). The proposed protocol is evaluated in a real environment. For this purpose, a test bed is developed using Raspberry Pi. The experimental results show that the proposed algorithm exhibits promising results in terms of network lifetime, average energy consumption, the packet delivery ratio, and average delay in comparison to an existing energy efficient routing protocol for SDWSN and a traditional source routing algorithm.

## 1. Introduction

A wireless sensor network (WSN) contains energy-constrained stationary or mobile wireless sensor nodes deployed in a dynamically varying environment. A sensor node consists of transmission, data processing, a power source, and multiple sensor units [1]. The WSN perceives and interacts with the physical world by revolutionizing the ways in application domains including environmental sensing, health, military defense, and habitat monitoring [2,3,4,5].

The majority of existing research work conducted in WSN focuses on proposing and developing low cost and low power networking solutions to perform collaborative and cooperative tasks under stringent computational and energy constraints. It is due to the fact that in various WSN applications, the wireless sensor nodes operate for a long duration without the replacement of their power units [6,7]. Therefore, the limitation of power capacity is considered extremely crucial while designing WSN strategies and solutions [8,9]. Routing is the core networking activity to route sensed information from sensor nodes to the sink. Hence, in such networks, the efficient utilization of node batteries must be guaranteed while designing routing strategies.

**Figure 1:**
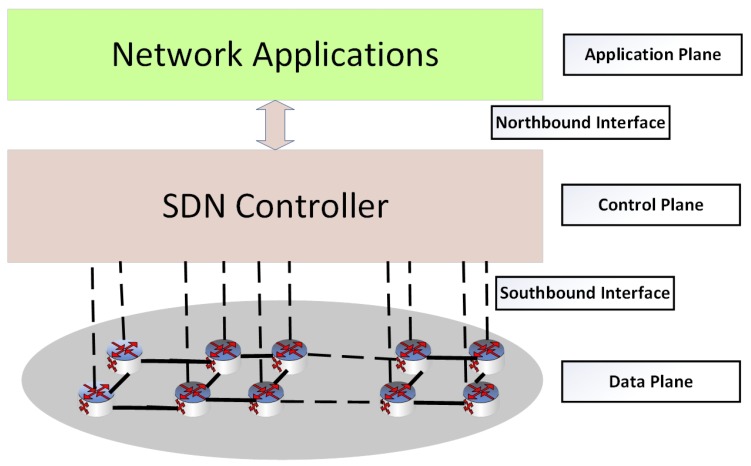
Software defined networking (SDN) architecture.

Software defined networking (SDN) is a revolutionary paradigm, promising to enable the evolution and dynamic management of traditional networks. It has a flexible network architecture that carries out the decoupling of network control that is programmable from the data plane [10,11,12]. In fact, the basic idea behind this concept is the efficient utilization and management of networking resources. In SDN, the network intelligence is logically centralized within the control plane, while the devices appear as packet forwarding elements within the data plane. The SDN uses a well-defined interface between various planes in the network. The control plane is responsible for routing and fault recovery while the data plane manages the packet delivery to devices. Considering the above-mentioned facts, it can be concluded that SDN can play a pivotal role in minimizing energy consumption in a WSN. SDN architecture is presented in Figure 1.

**Figure 2:**
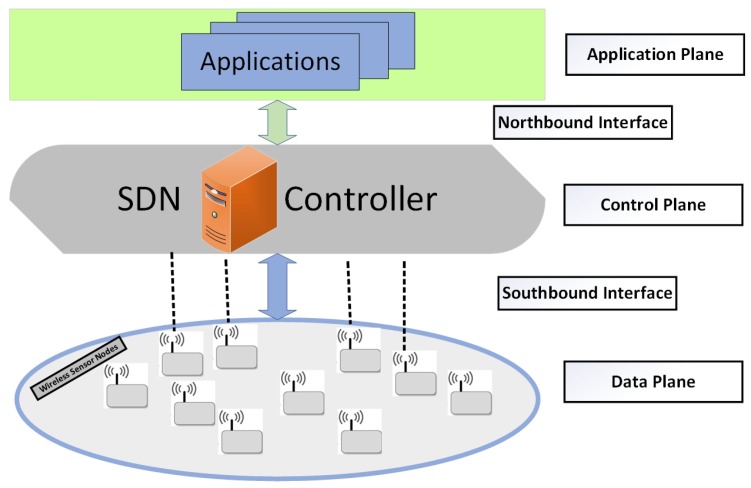
Software defined wireless sensor networks (SDWSNs) architecture.

In this paper, we integrate the concept of SDN to a traditional WSN, called software defined wireless sensor networks (SDWSNs) [13]. The architecture of a SDWSN is shown in Figure 2. We propose an SDN-enabled energy-aware routing protocol. In the proposed scheme, residual energy (R.E) and geographical proximity are the main parameters for the selection of the forwarder node. The proposed network architecture has two types of core nodes: Controller and regular nodes as shown in Figure 2. The underlying network is controlled by a controller that collects the data from regular nodes. Therefore, the aforementioned architecture plays an important role in managing the network more efficiently so as to minimize network energy consumption. The main contributions of this paper are listed below:

- Real-time implementation of SDN on hardware platform;
- The proposition and development of an energy aware SDN based multi-hop routing protocol (EASDN) for SDWSN;
- Development of a test bed useful for experimentation and analysis of SDWSN;
- The implementation of an existing energy efficient SDWSN routing protocol (called a traditional SDN [14]) and ad-hoc on-demand distance vector (AODV) routing protocol;
- A comparative analysis of an EASDN with a traditional SDN and AODV in real environment.

The remaining part of our paper is structured as followed: Section 2 describes related work and is divided into two parts, traditional WSN routing approaches and SDN based routing approaches. The energy consumption mathematical model is explained in Section 3. Section 4 provides details of the energy aware routing algorithm. The working of the algorithm is also demonstrated through figures. A real-time experimental setup, an experimental platform, a deployment scenario, and results are shown in Section 5. Finally, Section 6 concludes the paper and outlines future work.

## 2. Related Work

Each sensor node in a wireless sensor network (WSN) consists of a small battery for sensor node operations. The importance of the energy constraint factor in a WSN has become significant because of limited battery capacity. Routing is an important element in reducing the energy consumption of a WSN. Therefore, the need to reduce the energy consumption of a WSN is provided through many routing protocols. We divided the energy-efficient WSN routing approaches into following two different groups: Traditional WSN routing and software-defined networking based WSN routing.

### 2.1. Traditional WSN Routing Approaches

Traditionally, proactive and reactive routing is included in wireless network routing. Proactive routing (e.g., OLSR [15]) in each node depends on broadcast information, and all routing information (i.e., from the current node to all other nodes routing path information) is stored, which causes a memory overhead. That is why the high dynamic network does not adapt to active routing.

Reactive routing, such as an ad-hoc on-demand distance vector (AODV), has become an Institute of Electronics and Telecommunication Engineers (IETE) standard and was presented in 1999. It selects the routing path from source to destination by considering the number of hops [16,17]. AODV uses flooding to maintain and establish the route. Due to its flooding nature, the wastage of network resources is high.

Clustering protocols may help to aggregate the data by organizing the network in an inefficient manner. A low-energy adaptive clustering hierarchy (LEACH) [18] is a hierarchical protocol that transmits the data from nodes to cluster heads (CHs) to forward to the base station (sink). The CH role among nodes is played based on a predetermined probability by avoiding the fast depletion of CH energy. LEACH has two phases: The first phase is the cluster setup phase that selects the CHs to aggregate the information from its cluster and broadcast it to other nodes, and the second one is the steady phase that is used for the actual transmission of data. However, during the setup phase, LEACH only takes into account energy consumption when advertisements are received from CHs at every node. CHs do not show a good distribution because of variation in the number of CHs.

Moreover, in the LEACH protocol, a CH communicate with a sink node through a single hop in which a large amount of energy is consumed when the distance between CHs and sink is large. A heterogeneous protocol HEED (hybrid energy-efficient distributed) for clustering of wireless sensor network has been presented in [19]. Different levels of heterogeneity (i.e., 2-level, 3-level, and multi-level) are introduced to prolong network lifetime. In HEED, a cluster head is formed through the initial probability of every node based on its remaining energy that shows better performance in terms of network lifetime. A protocol named TEEN (threshold-sensitive energy-efficient sensor networks) has been presented for time critical based applications [20]. Often, data transmission is lesser as sensor nodes continuously sense the medium. A cluster sensor directs a small change in the soft threshold (ST) to trigger the node that has switched on its transmitter, and a hard threshold (HT) shows a value of sensed attribute. The disadvantage of this scheme is that the user does not receive any data because the nodes will not communicate when thresholds are not received. It also offers the implementation scheme of threshold-based functions [20] and suffers from the complexity in forming a cluster at multiple levels. In [21], zone probabilistic routing (ZPR) is proposed to reduce energy consumption and enhance network lifetime. The data packets are sent randomly routed from source to destination through any path within the defined routing zone. In ZPR, Four Probability Distributions (4PD) are used to define the probability distribution that is completely controlled via a set of control parameters (residual energy, perpendicular distance, direction, and transmission distance).

The issue of load balancing in WSN clustering also becomes critical that leads to increasing the WSN energy consumption and is addressed by some authors [22,23] that consider the hop distances for the clusters. A scheme named energy-delay index for trade-off (EDIT) in [24] is proposed to select the CH for the optimization of both delay and energy. In [25], a delay award energy efficient Routing (DERM) protocol was proposed to achieve the energy efficiency of WSN with minimum delay. DERM utilizes a location-based greedy forwarding technique for sending relay packets to the destination within a delay constraint.

In [26], the reinforcement learning based topology control mechanism (LBLATC) is proposed to reduce the energy consumption of a WSN by automatically adjusting the transmission range. Each sensor node selects the neighbor and adjusts the transmission range through the learning process. In using a LBLATC protocol, each node selects the shortest transmission range that results in enhancing the network lifetime. In WSa N, security is also one of the main issues with sensor node energy consumption. Each sensor node of WSNs plays a critical role to protect the network from attacks. The WSNs can resist against attacks by focusing on a self-protection mechanism. In [27], the authors proposed an algorithm that is based on learning automaton to preserve the protection of sensors. To protect network nodes, the proposed technique tries to activate the minimum number of nodes. The authors claim that their algorithm performs better in terms of a number of active node ratio, and energy consumption. In [28], a distributed border surveillance (DBS) algorithm is proposed for security purposes that maximizes the number of barriers and minimizes energy consumption. The DBS algorithm is based on learning automaton that assures barrier coverage by finding the best nodes in terms of security. A data transmission framework in [29] is designed to facilitate packet loss information for a suitable industrial environment. However this WSN framework is not suitable in a real industrial environment due to following the environment’s properties such as diversity, a strict need for data transmission, a harsh application environment, and so on.

### 2.2. SDN Based WSN Routing Approaches

A common issue in previous research is that simple models and concepts have been put forward with simulations that are simple or not even realized. There is no systematic realization or explanation as detailed on algorithms of a controller for SDN routing is relatively vague [30].

Recently, many prototypes are practically implemented because of wireless networks as explained in [31]. SDWSNs have enabled the programmable control and virtualization of equipment in networks through the decoupling of data and the control plane [32]. In a logically centralized controller, the control intelligence is implemented after taking it out of the data plane devices that use the standard interfaces to interact with data plane devices. The software programs are performed by network operators to optimize network resource usage and automatically manage data plane devices. This architecture is used to ensure up-to-date control schemes for the future management of SDWSNs [33]. In [34], a SDN based published/subscriber system (SDNPS) is designed for a load balancing issue that helps to reduce end-to-end latency. A framework for a SDWSN was proposed by Jayashree in [35] where the forwarding is executed by sensor node only. The energy consumption is reduced through the implementation of a controller as a base station in this framework.

The sensor nodes in a SDWSN reconfigure their properties and functionalities dynamically through the loading of multiple programs on demand regarding real-time sensing requests. The issues mentioned above are tackled by emerging SDWSNs that act as a compelling solution. A variety of sensing nodes are undertaken by considering the activated programs because such nodes are equipped with different types of sensors.

The energy consumption of transmitters is minimized by designing an efficient SDWSN as proposed in [36]. In this article, the energy transmitters are used to transmit energy to sensor nodes. For an optimal replacement of energy transmitters, a trade-off is made between maximum energy charged and fair energy distribution. It may not result in energy efficient routing protocols. An energy-efficient routing algorithm for SDWSN has been proposed in [37] by dynamically assigning and selecting the different types of tasks and control nodes respectively. The particle swarm optimization method was utilized to select the selection of some control nodes in SDWSN. The nodes assign the tasks on the base of their residual energy. Thus, there is a difficulty in determining the precise number of required control nodes as well the number of nodes under one particular control node.

Duan [38] proposed a framework called Improved SDWSN for a SDN-based network to enhance network reliability. The proposed framework helps to resolve the network management of WSN in the Industrial Internet of Things (IIoT) and improves network reliability. They also claimed to address node failure issues, especially related to energy consumption, but they do not use any energy aware algorithm to control the energy consumption of nodes.

In [14], an energy efficient routing algorithm is proposed for a SDN-based WSN network. The authors assume that the controller knows the initial data of all network nodes and the controller establishes a flow table based on distance. They assume that the controller can access each node by hop. After establishing the flow table, the controller sends to each node directly, which is impractical in a large network. An SD-EAR energy aware routing algorithm [39] is designed to reduce the flooding and broadcasting issue of WSN network. The authors divide the network into a different zone and each zone is handled by the SDN controller, but the selection of the zone head is vague.

A routing algorithm is proposed for a SDN network in [40]. The controller collects the node information through multihop communication. A controller generates the flow table based on hop count and energy. The hop count may consume more energy if the distances between two nodes are large and the nodes will die quickly which can reduce the network lifetime. The authors use the OPNET for simulation, and the results are compared with optimized link state routing (OLSR) and AODV.

In [41], the authors proposed two algorithms (greedy and global greedy) based on a SDN for optimizing the power management of chassis and line-cards on a network level. A 0–1 integer linear programming is used to minimize power utilization. The proposed algorithm shifts the traffic from higher to lower traffic branches to balance energy consumption.

Mostly, the previous work does not give a better solution for real-time WSN application. To resolve these issues, we present the energy aware routing algorithm that is used for real-time applications in the next section and also show the real-time deployment of wireless nodes (i.e., Raspberry Pi) for experimental work.

## 3. Energy Consumption Mathematical Model

We considered the first order radio model [42] in our experiments for the energy consumption calculation used for data communication. For path losses calculation, two channel models are used in this energy consumption model: One is a free space model and the other is multipath fading [14,37]. The selection of any model for calculating the energy consumption is made on the basis of distance between transmitter and receiver. The free space model is used when the distance between transmitter and receiver is less than or equal to the threshold $S_{0}$, otherwise multipath fading model is selected for computing energy consumption. The energy consumption for transmission of each packet is calculated by:

(1) $$E_{Tx}\left(l_{bits},S\right)=\left\{\begin{array}{cc} l_{bits}*E_{fs}*S^{2}+l_{bits}*E_{elec} & S\leq S_{0}\\ l_{bits}*E_{mp}*S^{4}+l_{bits}*E_{elec} & S>S_{0} \end{array}\right.$$

where $E_{Tx}$ is the energy consumption for transmission, $l_{\mathrm{bits}}$ is a length of packet (i.e., number of bits in each packet), and the distance between transmitter and receiver is *S*. $E_{\mathrm{elec}}$ is the energy consumption due to the transmitter and receiver circuit to process the data before sending or receiving while $E_{\mathrm{fs}}$ and $E_{\mathrm{mp}}$ are dependent on transmitter amplifier model. $S_{0}$ is the transmission threshold which is defined as:

(2) $$S_{0}=\sqrt{E_{fs}/E_{mp}}.$$

Energy consumption during reception of data packet is calculated as:

(3) $$E_{Rx}=l_{bits}*E_{elec}.$$

## 4. Energy Aware Software Defined Network (EASDN) Routing Algorithm

In a WSN, each sensor node has a different communication range to target and send data periodically. In a conventional WSN, sensors broadcast the control messages and data information periodically which leads to energy waste. The control plane is separated from the data plane through SDWSN. In the initial phase of SDWSN, the controller needs to collect the topology information from the underlying network because the control plane is responsible for generating the topology and resource allocation in SDWSN. Software defined networking facilitates to the user, but it has some cost that can be manageable during the topology changing phase as compared to a traditional WSN. SDWSN manages the network resources efficiently by the controller due to its global view, which reduces the sensor nodes’ energy consumption significantly. This paper presents the design of an energy aware software defined network (EASDN) routing algorithm that is used to balance the energy consumption of sensor nodes to prolong network lifetime.

In our energy aware approach, three phases are included: Neighbor discovery phase, status data collection phase, and the operational phase. The detailed algorithm is shown in Algorithms 1, 2, and 3.

**Figure 3:**
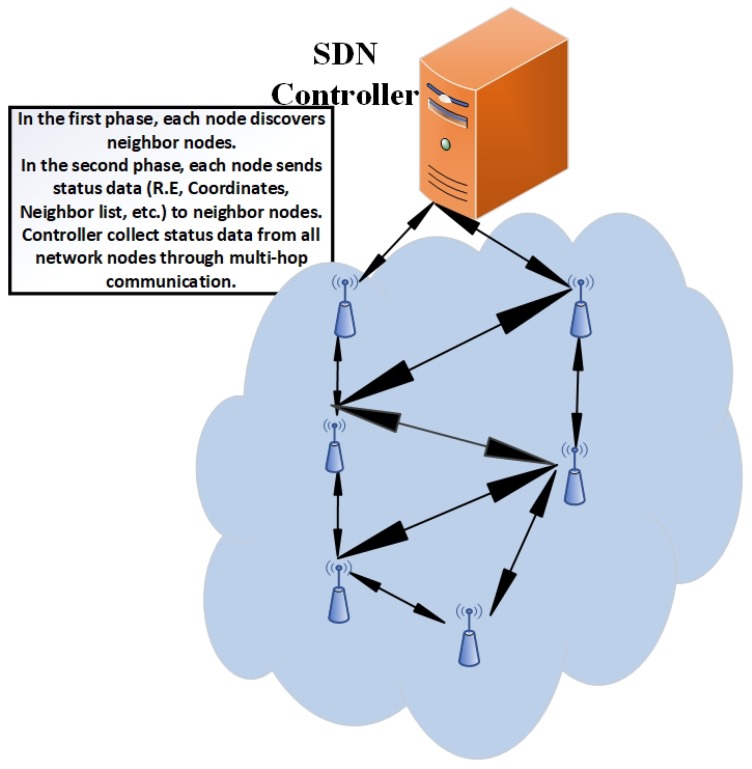
Neighbor discovery and status collection phase.

In the neighbor discovery phase, each node discovers its neighbor through broadcasting. As shown in Algorithm 1, each node broadcasts a ’Hello’ message and also receives the ’Hello’ message from parallel neighboring nodes. If a sensor node finds any new neighbor (which neighbor ID does not exist into the neighbor list), it adds into the neighbor list. Otherwise, it discards the neighbor’s ’Hello’ message. The first phase stops after the time threshold or when the maximum number of nodes is reached. After the discovery phase, the status data collection phase starts. The whole procedure is given in Algorithm 2. In the status data collection phase, each node shares its own data with neighboring nodes. When neighbor node receives the status data, it forwards to neighbors excluding that neighbor from which the node receives data and the whole procedure continues up to controller neighbor nodes. Hence, the controller collects the data of the whole network through multi-hop communication up to a specific time threshold or up to a maximum number of nodes reached. Figure 3 describes the phase one and phase two.

**Figure 4:**
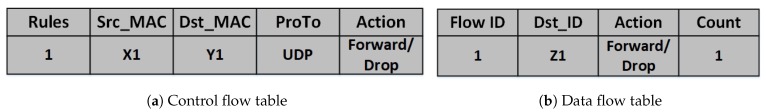
An example of a flow table.

**Figure 5:**
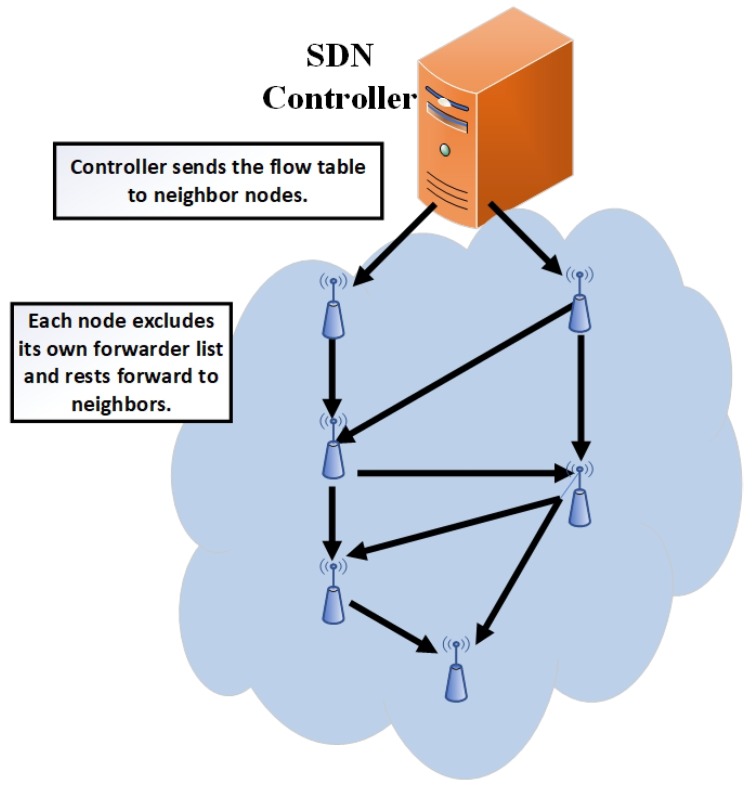
Received flow table.

After collecting the whole network data, the controller operates the energy-aware algorithm and generates the flow table according to the distance from a controller and residual energy. The example of flow table is shown in Figure 4 and the procedure of the controller operational phase is given in Algorithm 3. The controller sends the control flow table to neighboring nodes (that are reachable to the controller) which extracts the desired data from the flow table and forwards the rest to neighboring nodes. Therefore, the whole network nodes receive the flow table through multi-hop communication. Figure 5 shows the forwarding of the flow table scenario.

**Figure 6:**
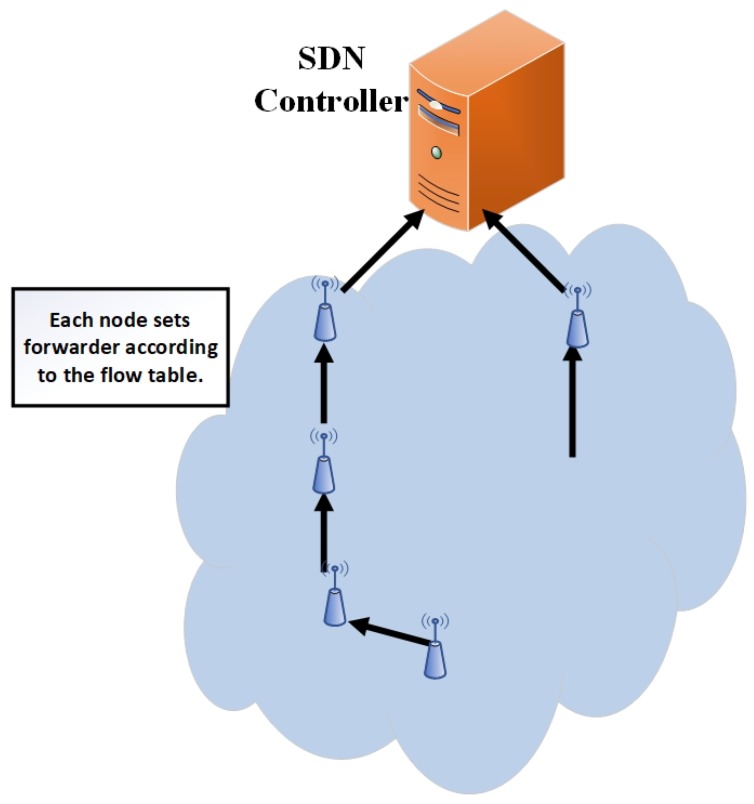
Routing path established.

After receiving the flow table from the controller, the operational phase starts. Each node follows the controller flow table instruction and establishes the routing path according to the flow table, which is shown in Figure 6.

**Algorithm 1** Neighborhood discovery phase
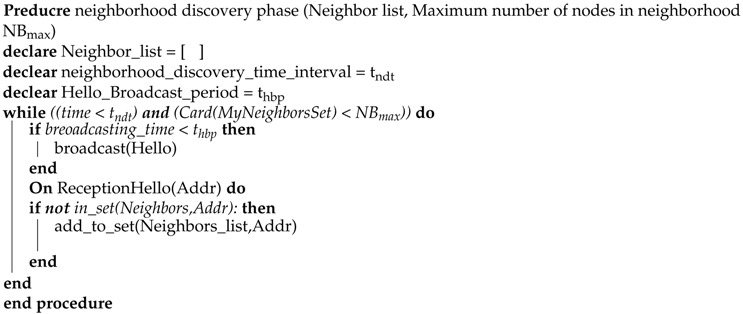


**Figure 7:**
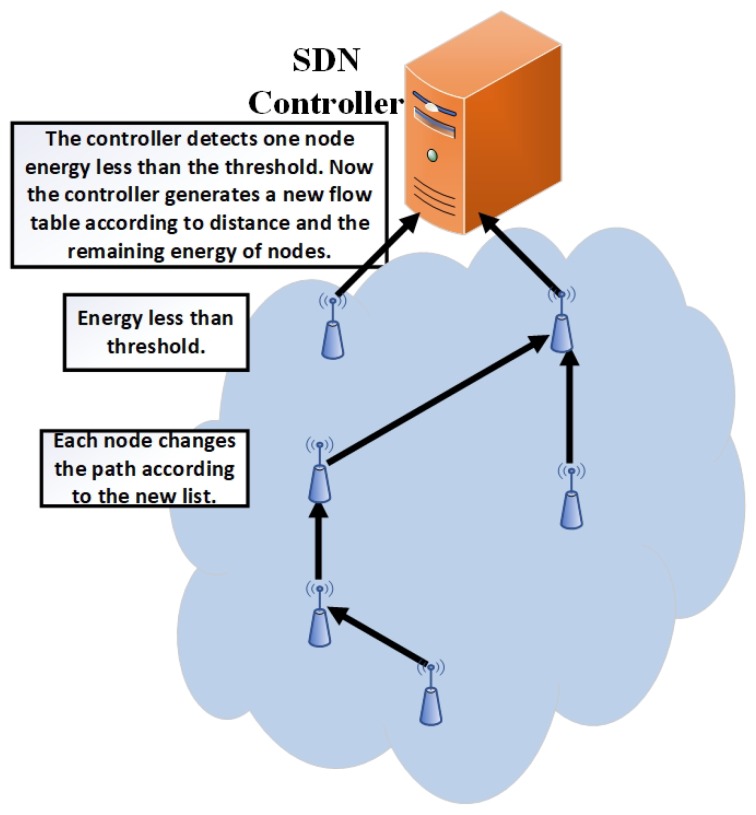
Updating phase 1.

During the operational phase, the network nodes follow the same flow table instructions for sending node data (e.g., after each second generate packet) to the controller until any node dies or the node energy level is lower than the defined threshold. When the controller observes that any node energy goes less than a defined threshold, then it generates a new list according to the residual energy and distance. First, it will check the shortest distance between neighboring nodes to select the forwarder node from the short distance neighbor list, which has high residual energy. The whole procedure continues until all network nodes die. Its scenario is shown in Figure 7. During the operational phase, if any node becomes disconnected due to hardware failure, the neighbor node will intimate to the controller about the disconnected node.

**Figure 8:**
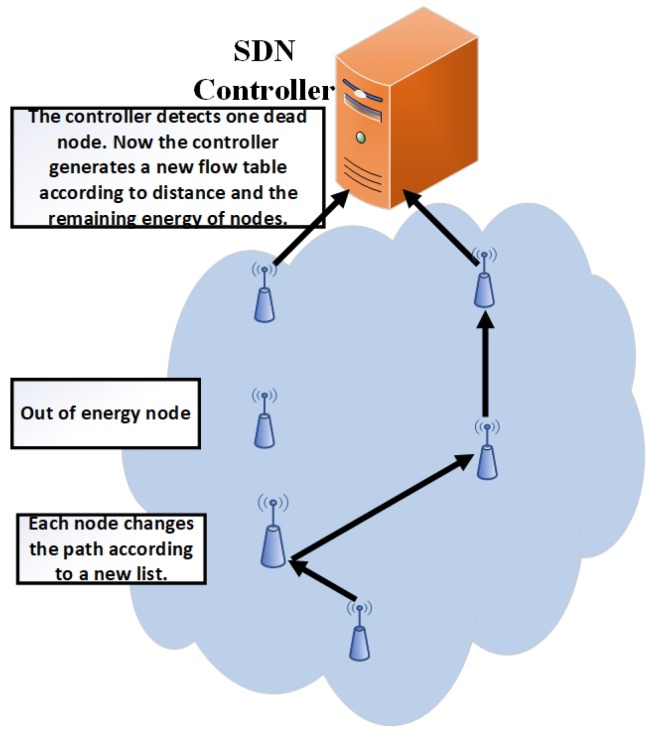
Updating phase 2.

If the controller observes that any node runs out of energy or is disconnected due to any reason (e.g., hardware failure) then it removes the detected node related data from the status list and follows the same procedure as in updating phase 1 as shown in Figure 8 and described in Algorithm 3. The controller gets the information of residual energy of each node from the sensor node traffic, which is also collected at the controller (the controller controls the network as well as collects the node data packets). The whole algorithm detail is given in Algorithms 1–3.

**Algorithm 2** Status data collection phase
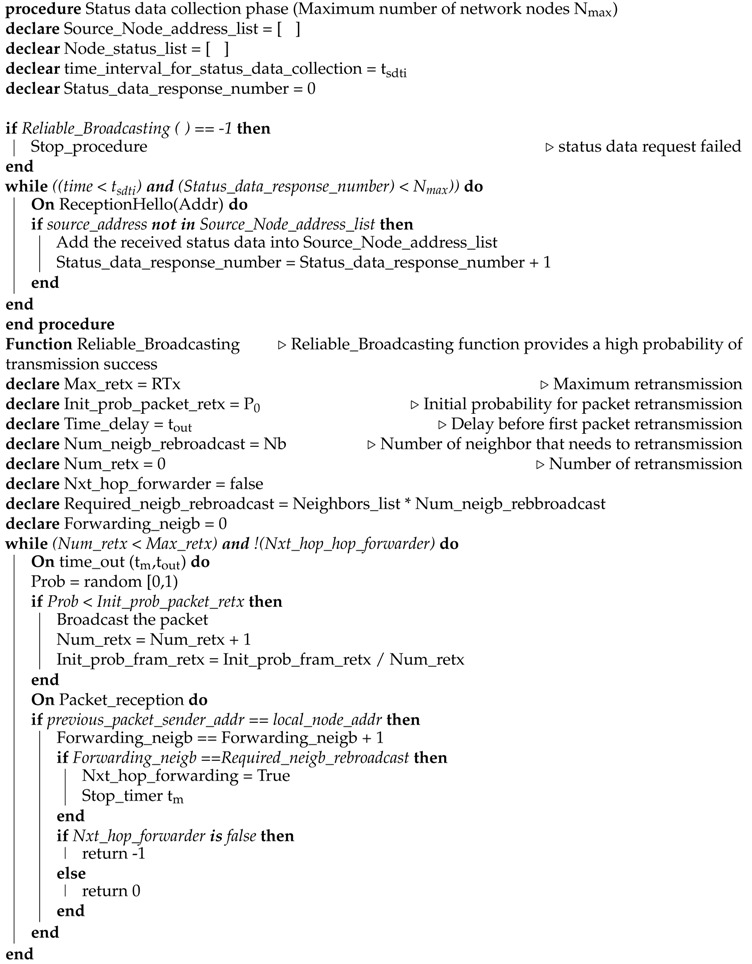


**Algorithm 3** Controller operational phase
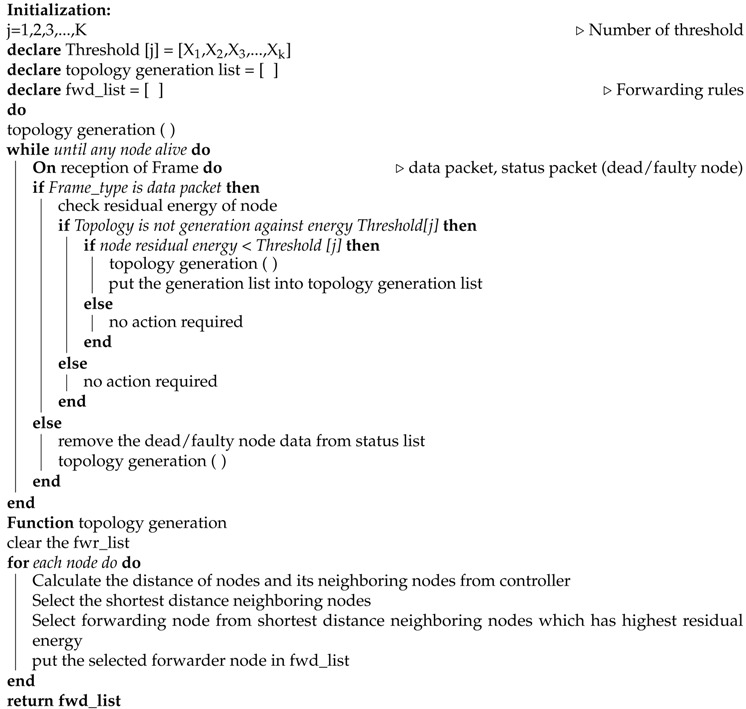


## 5. Experimental Setup and Results

### 5.1. Experimental Setup

**Figure 9:**
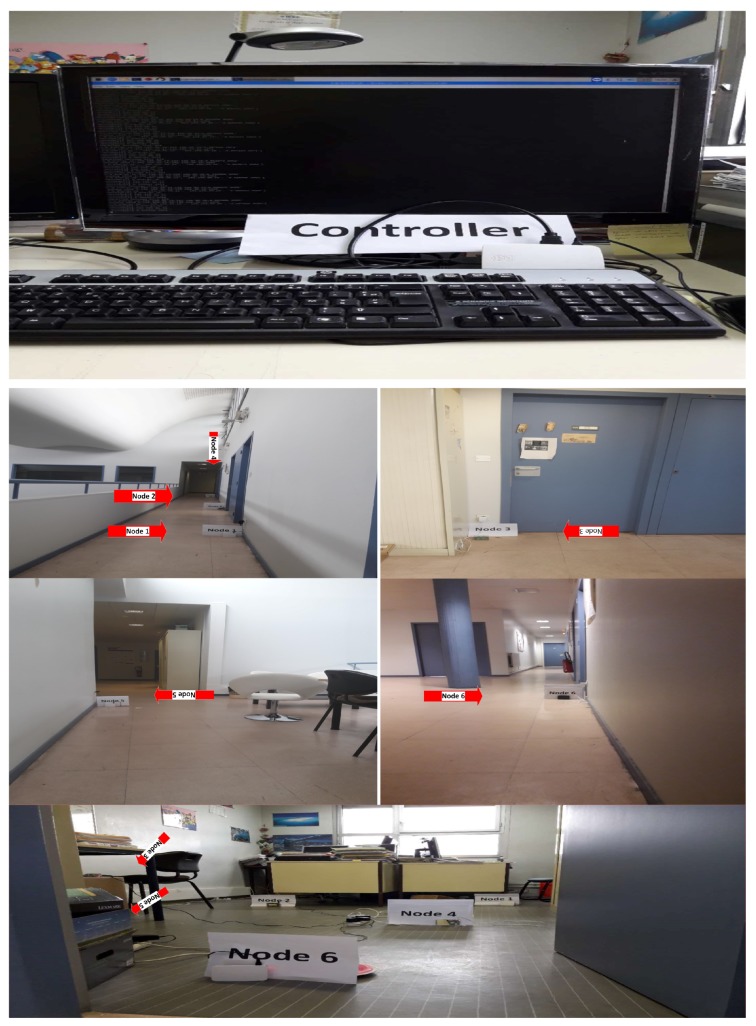
Real-time nodes deployment on the fourth floor of IRIT-1 building.

**Figure 10:**
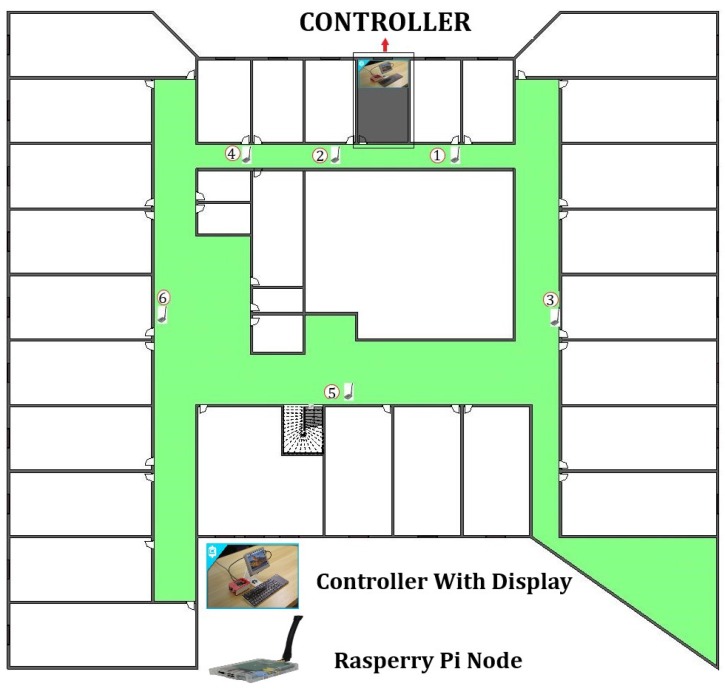
Raspberry Pi nodes deployment structure on the fourth floor of IRIT-1 building.

In this section, we will explain the experimental results. First we develop the ad hoc network using a 802.11ac wireless LAN. Each node can connect with neighbor nodes through a wireless ad hoc connection. In our experimental scenario, the node’s communication is real-time with simulation-based energy consumption calculation. For energy consumption measurement, we used a simulation because Raspberry Pi cannot measure the directly residual battery capacity. It needs an external chip (i.e., MoPi) which can measure it. The energy consumption model is already explained in Section 3 as to how to calculate the energy consumption and the simulation parameters used to calculate the energy consumption are given in Table 1. We deployed six Raspberry Pi 3 as sensor nodes and one node as a controller in two different areas at the fourth floor of IRIT-1building, University Paul Sabatier Toulouse as shown in Figure 9, which has a 30 m × 38 m area and the second deployment was in one room which has a 3 m × 10 m area dimension. Each raspberry pi connects with neighbor nodes which are accessible in his range through ad hoc connection and each pi node sends a data packet up to a controller though multihop. The deployment scenario is shown in Figure 10.

### 5.2. Experimental Platform

We implemented our experiment by using Python 3.0 and tested it on Raspberry Pi 3 that is low cost and low-powered with a credit size single-board computer. Raspberry Pi is used for both the sensor node to transmit the data and controller that controls the whole network as well as collects the data of sensors. Recently, it has become very popular because of its use in robotics projects, WSN, and cloud computing applications [43,44,45]. In our experimental work, we used Raspberry Pi B+ model. It comes with a powerful 1.4 GHz 4 x Cortex-A53 CPU and runs an ARMv8 microcontroller with 1GB RAM [36]. It supports various operating systems including a Debian Linux-based OS that is used to optimize the Raspberry hardware and also recommended by the Raspberry Pi foundation. The Raspberry Pi B+ model has good specifications when compared to other models. RPi B+ model also contains 802.11ac wireless LAN card that is used for wireless communication (e.g., wireless ad hoc connection). It is also deployed as an intelligent sensor node [43,44,46] in many WSN networks and boosted by a micro SD card.

### 5.3. Evaluation Metrics

The experimental results are based on the following metrics:

#### 5.3.1. Network Lifetime (LT)

It is a time duration in which the network remains operational. We considered two definitions to calculate the network lifetime:

- The time until the first node runs out of energy;
- The time until the last node (sink is not reachable) runs out of energy.

#### 5.3.2. Packet Delivery Ratio (PDR)

It is the percentage of packets successfully delivered at the destination. *TPTx* is the total number of packets transmitted from all network nodes while TPRx accounts the total number of packets received at the destination during experimental time.

(4) PDR=(TPRx/TPTx)×100.

#### 5.3.3. Number of Alive Nodes (NAN)

It is the summation of the number of nodes that are alive after each round (time step). One round is completed when each node generates the packet after a specific period (i.e., 1 s, 2 s).

#### 5.3.4. Average Energy Consumption Per Bit (ECPB)

It is a summation of all nodes initial energy, divided by the total number of received bits.

(5) ECPB=∑(Initialnodeenergy)/Totalreceivedbits.

#### 5.3.5. Average Delay (AD)

The summation of delay which is faced by all packets to reach up to the destination divided by total transmitted packets. PD is the packet delay which is faced by the packet and TTP is total number of transmitted packets.

(6) AD=∑PD(ttp)/TTP.

ttp∈TTP

### 5.4. Results and Discussion

**Figure 11:**
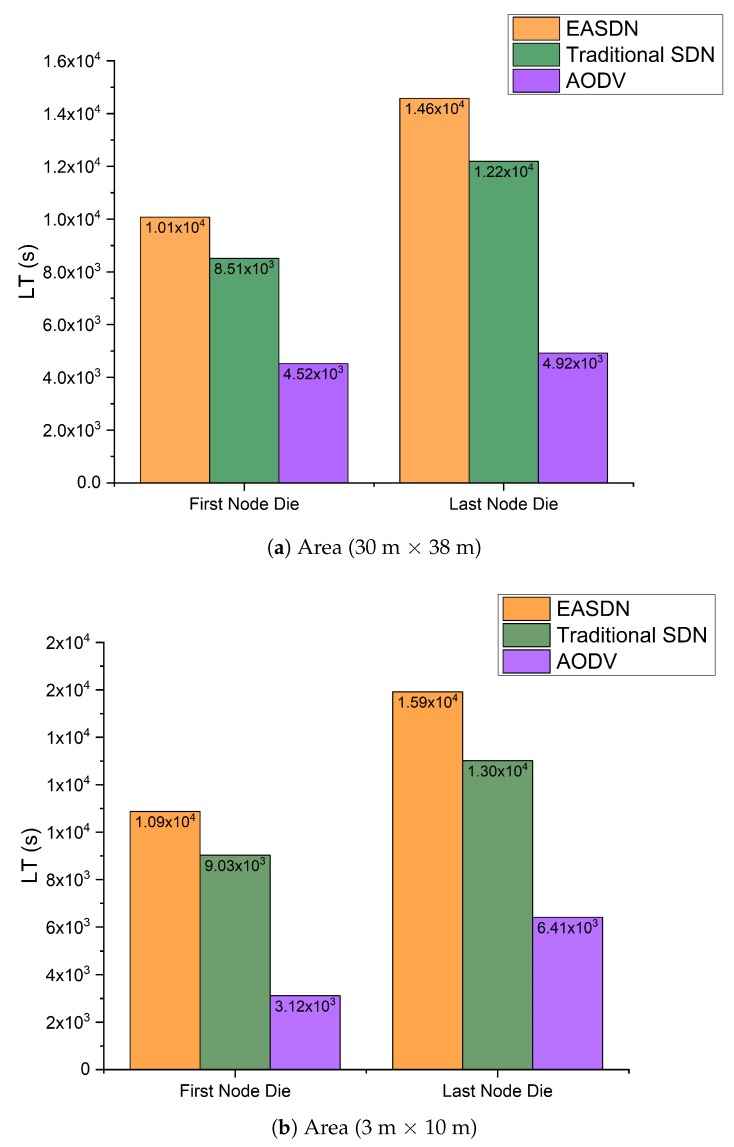
Network lifetime.

**Figure 12:**
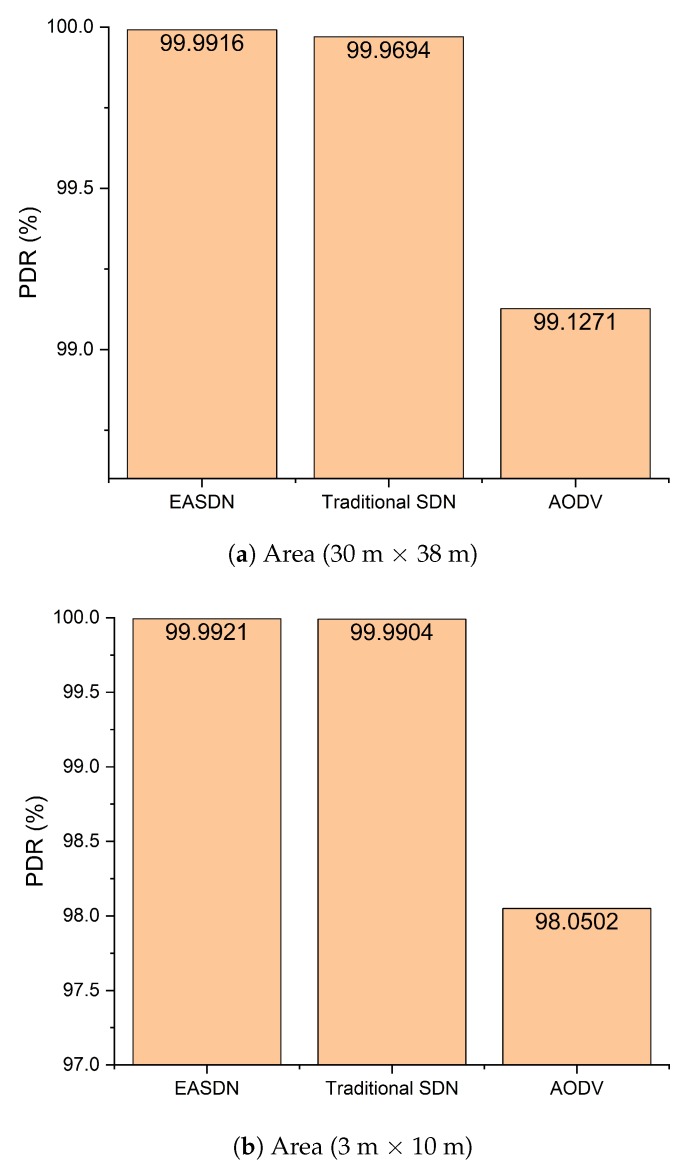
Packet delivery ratio.

**Figure 13:**
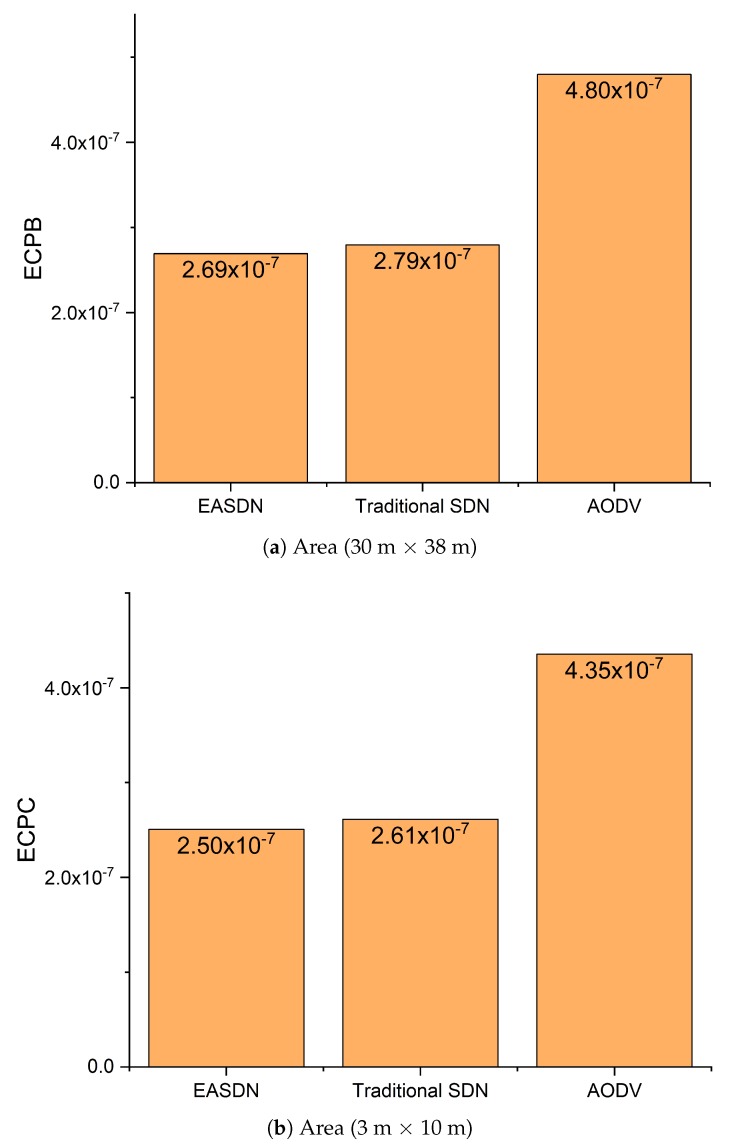
Energy consumption.

**Figure 14:**
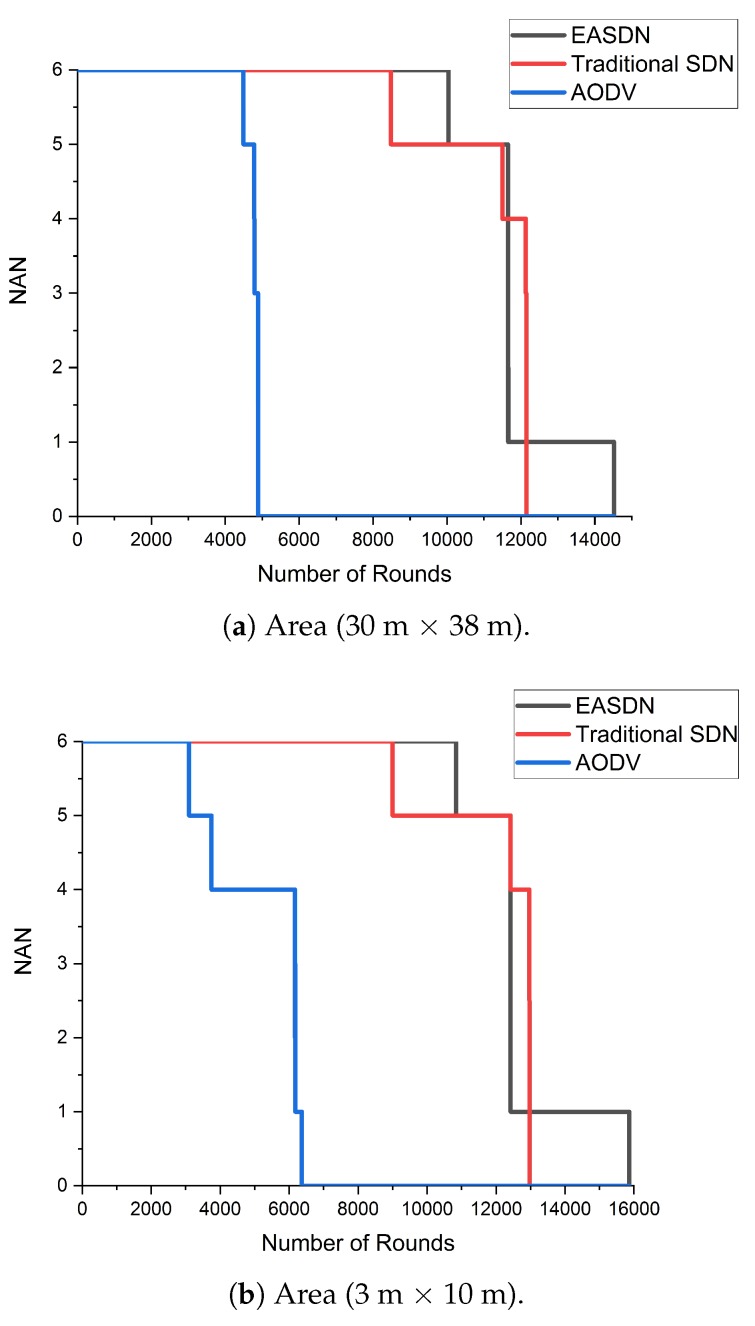
Alive nodes vs number of rounds.

**Figure 15:**
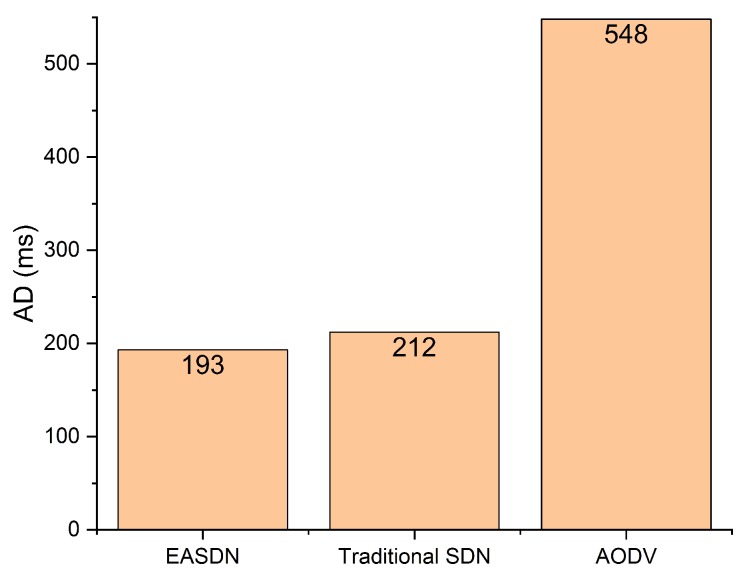
Average delay (30 m × 38 m).

We performed our experiment in two different areas: Firstly, the nodes were deployed on the fourth floor of IRIT-1 in a 30 m × 38 m area and then the nodes were deployed in room that has an area dimension of 3 m × 10 m. We took different metrics and compared the EASDN proposed algorithm results with the traditional source routing algorithm based on SDN (traditioanl SDN) and AODV as shown in Figure 11, Figure 12, Figure 13, Figure 14 and Figure 15.

In Figure 11, the lifetime of both dimensions is shown. The network lifetime of an EASDN is higher than both a traditional SDN and AODV. In an EASDN, the proposed algorithm balances the energy consumption of each node by defining the energy threshold of the node, which is used as a forwarder. In the EASDN algorithm, the controller changes the forwarder node if it observes any forwarder is less than a threshold. However, in a traditional SDN, once the path is established then it changes the forwarder node when the controller observes any node has run out of battery whereas in an AODV, each node uses broadcasting to setup the path up to the destination. Firstly, it needs the destination address in each packet, however in a SDN and EASDN, each node is responsible for sending the packet up to one hop, then the next hop has a responsibility to route the packet towards the feasible path. Secondly, an AODV uses broadcasting when it needs to establish a new path which consumes high energy. Therefore in the graphical representation, we can see that if we consider the network lifetime definition according to the first node death then the EASDN lifetime is 10,074 s when the area is 30 m × 38 m, which is higher than both a traditional SDN and AODV lifetime, which are 8509 s and 4522 s respectively. If we follow the second definition of a network lifetime, the network lifetime of EASDN is 14,574 s, which is also higher than a SDN network lifetime and AODV lifetime, which are 12,193 s and 4922 s respectively as shown in Figure 11a. The experimental results show that an EASDN enhances the network lifetime 18% to 20% when compared to a traditional SDN.

In the second scenario, we changed the area dimension in order to see the effect on network lifetime. The results are similar to those in the 30 m × 38 m dimension area, but in the current scenario, the experimental area is smaller, which means the distance between nodes is also smaller. Therefore, the nodes consume less energy which leads to higher network lifetime. We can observe from Figure 11b that the network lifetime is higher in a 3 m × 10 m dimension than a 30 m × 38 m dimension. As shown by the results, an EASDN increases network lifetime 20% to 22% when compared to a traditional SDN.

From Figure 12, we can observe the packet delivery ratio (PDR). An EASDN has a higher PDR thana traditional SDN and AODV. In a traditional SDN, some packets were lost due to network congestion. It continues the same path until any node dies and in an AODV, the packets were lost due to its broadcasting nature. It uses broadcasting to establish the network path whenever any node needs new path. Therefore, if any forwarder node dies then it establishes a path through broadcasting. During the broadcasting time period, the number of data packets cannot reach up to the destination path during the establishing period, and as a result the underlying network packets (e.g., the underlying network goes to disconnect when the relay node dies) become lost. Furthermore, an AODV could not establish a stable path when compared to the SDN based network because it does not have a global view to manage the network efficiently. Therefore, the AODV has the lowest PDR performance. However, the performance of the EASDN is higher than both a traditional SDN and AODV.

As shown in Figure 12a, the PDR of an EASDN is 99.9916%. However, the PDR of a traditional SDN is 99.9694% and an AODV is 99.1271%. So in terms of PDR, the EASDN is also performing better than a traditional SDN and AODV.

In the second scenario, the PDR of the EASDN is also better than a traditional SDN and AODV as shown in Figure 12b.

Figure 13 shows the average energy consumption per bit. In the first experimental scenario, the EASDN has lower energy consumption per bit than a traditional SDN and AODV, because the EASDN balances the energy consumption of each network node. Therefore, in an EASDN each node is able to send more numbers of packets when compared to a traditional SDN and AODV. In an AODV, each time node needs to broadcast to establish the path, which leads to more energy consumption. In Figure 13a, we can see the energy consumption per bit of an EASDN is 2.6919 × 10${}^{-7}$ whereas a traditional SDN and AODV consume 2.7949 × 10${}^{-7}$ and 4.7993 × 10${}^{-7}$ , respectively.

In the second scenario, the EASDN energy consumption per bit is also lower than a traditional SDN and AODV as shown in Figure 13b. The description is similar to the one above for the first scenario.

In Figure 14, we compared the network alive nodes with different algorithms. Here we used only the second definition of life time (e.g., the time until the last node runs out of energy.) As shown in Figure 14a, an EASDN algorithm has more number of rounds than traditional a SDN and AODV. An EASDN is an energy aware algorithm. It changes the path of the forwarder node when it observes any node has less energy when compared to neighbor nodes, so it manages the network concerning residual energy. However, in a traditional SDN, it could not balance the energy consumptiondue to the fixed pathand it also uses only short distances for establishing the routing path. Therefore, it may use the forwarder node which has a low residual energy when compared to the neighboring node that leads to disconnect the underlying nodes. In an AODV, it uses the hop count for establishing the path, but the AODV could not provide the optimal hop count because it does not have a global view and sometimes the hop count is higher, which leads to consuming more energy. As shown in Figure 14a, when the EASDN algorithm is implemented, it gives more than 14,000 rounds however in other traditional SDN and AODV algorithms, they give approximately 12,000 and 6000 rounds.

In the second scenario, when the area dimension is smaller, each algorithm gives a number of rounds as shown in Figure 14b that also show a similar behavior described in the first scenario.

Figure 15 shows the average delay comparison between our proposed algorithm and a traditional SDN and AODV. The proposed algorithm gives a better performance in terms of average delay because it does not use the broadcasting to develop the routing path like in the AODV. In the AODV, each node uses the broadcasting, which takes a long time for establishing the path. Sometimes it chooses a long path in terms of distance because it counts the least number of hops even if the distance between the two nodes is too large therefore, it leads to increase the transmission time from source to destination. As shown in Figure 15, the proposed algorithm has 193 ms average delay where the traditional SDN and AODV have 212 ms and 548 ms, respectively.

## 6. Conclusions and Future Work

SDN-enabled WSN provides a dynamic, flexible, and easy to manage paradigm to revolutionize the traditional WSN. Consequently, in this paper we utilized the SDN potential to reduce energy consumption in a WSN by proposing an energy efficient routing protocol. The main purpose of the proposed protocol was to increase network lifetime. The proposed scheme was implemented in a real environment. The evaluation results show that an EASDN enhanced network lifetime from 18% to 22% in comparison to a traditional SDN and AODV. Moreover, it also decreased the average delay and improved packet delivery ratio.

In future, we intend to implement and evaluate the proposed scheme on a large scale scenario using a simulations. We also plan to implement the machine learning based routing algorithm for optimizing the energy consumption of network nodes to enhance the network lifetime.

## Figures and Tables

**Table 1:** Energy parameters.

Parameter	Value
Initial energy	1 J
E${}_{\mathrm{elec}}$	50 nJ/bit/m${}^{2}$ [14]
E${}_{\mathrm{fs}}$	100 pJ/bit/m${}^{2}$ [14]
E${}_{\mathrm{mp}}$	0.0013 pJ/bit/m${}^{4}$ [14]
Data packet size	296 bit
